# Recent Advances in Nano-Engineered Thermochemical Energy Storage Materials: Morphologies, Characteristics, and Performance

**DOI:** 10.3390/nano15191476

**Published:** 2025-09-26

**Authors:** Zhu Jiang, Wenye Li, Bohao Peng, Shifang Huang, Xiaosong Zhang

**Affiliations:** 1 School of Energy & Environment, Southeast University, Nanjing 211189, China; 2 Engineering Research Center of BEEE, Ministry of Education of China, Nanjing 210096, China

**Keywords:** thermochemical energy storage, nanomaterials, sintering, agglomeration, reaction kinetics, cycling stability

## Abstract

Thermochemical energy storage (TCES) has gained significant attention as a high-capacity, long-duration solution for renewable energy integration, yet material-level challenges hinder its widespread adoption. This review for the first time systematically examines recent advancements in nano-engineered composite thermochemical materials (TCMs), focusing on their ability to overcome intrinsic limitations of conventional systems. Sorption-based TCMs, especially salt hydrates, benefit from nano-engineering through carbon-based additives like CNTs and graphene, which enhance thermal conductivity and reaction kinetics while achieving volumetric energy densities exceeding 200 kWh/m^3^. For reversible reaction-based systems operating at higher temperatures (250–1000 °C), the strategies include (1) nanoparticle doping (e.g., SiO_2_, Al_2_O_3_, carbonaceous materials) for the mitigation of sintering and agglomeration; (2) flow-improving agents to enhance fluidization; and (3) nanosized structure engineering for an enlarged specific surface area. All these approaches show promising results to address the critical issues of sintering and agglomeration, slow kinetics, and poor cyclic stability for reversible reaction-based TCMs. While laboratory results are promising, challenges still persist in side reactions, scalability, cost reduction, and system integration. In general, while nano-engineered thermochemical materials (TCMs) demonstrate transformative potential for performance enhancement, significant research and development efforts remain imperative to bridge the gap between laboratory-scale achievements and industrial implementation.

## 1. Introduction

The global transition to renewable energy is accelerating, yet the intermittent nature of solar and wind power remains a critical challenge for grid stability and energy reliability [1]. According to the International Energy Agency (IEA), renewable energy sources are expected to account for 60% of the total generation by 2050 [2]. The inherent intermittency and variability of renewable energy generation pose substantial challenges to grid stability, creating a critical need for effective energy storage technologies [3,4,5,6]. Unlike electrochemical storage such as batteries and supercapacitors, which excel in having a high power density and rapid response [7], thermal energy storage (TES) features low-cost, long-duration stability, and broader temperature adaptability, making it more suitable for large-scale heat recovery, industrial process integration, and seasonal energy storage [8,9,10,11]. The global thermal energy storage (TES) market, which reached a valuation of USD 4.5 billion in 2022, is anticipated to expand significantly with a projected 10.3% compound annual growth rate (CAGR) through 2030 [12]. 

**Figure 1:**
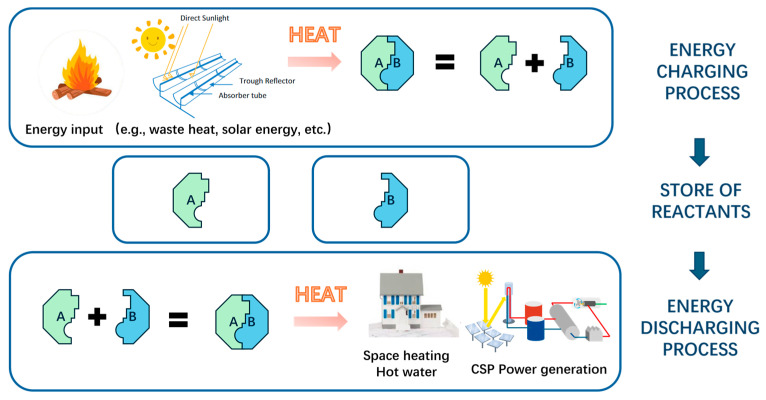
Schematic illustration of thermochemical energy storage.

Thermochemical energy storage (TCES), a method of storing thermal energy by sorption or reversible chemical reaction, offers distinct advantages over sensible and latent heat storage, including higher energy density (~500 kWh/m^3^ [13]), minimal thermal losses over long periods, and the ability to store energy indefinitely until needed [14,15,16]. These features make TCES particularly suitable for seasonal energy storage, waste heat recovery, and integration with concentrated solar power (CSP) plants; see Figure 1. Despite its potential, the widespread deployment of TCES has been hindered by material-level challenges. Conventional thermochemical materials (TCMs) includes sorption-based (e.g., salt hydrates, zeolites) and reversible reaction-based (e.g., hydroxides, carbonates, redox oxides) systems [17,18]. Sorption TCMs, which operate at low-to-medium temperatures (<250 °C), are ideal for building heating and cooling applications, but their deployment is often limited by deliquescence, low thermal conductivity, agglomeration, and cycling instability [19,20,21,22]. Reversible reaction-based systems, e.g., Ca(OH)_2_/CaO and Co_3_O_4_/CoO, can store energy at high temperatures (300–1000 °C), making them suitable for industrial processes and CSP integration [13,23,24,25,26]. However, these materials suffer from sintering, incomplete reversibility, low thermal conductivity, and slow kinetics and cycling instability, which reduce their large-scale deployment.

Nanomaterials, with their high specific surface area, excellent thermal conductivity, and tuneable surface chemistry, offer promising solutions to these limitations [27,28,29,30,31,32]. For instance, nano-enhanced phase change materials and sensible storage materials (e.g., molten salt) have been extensively studied for their ability to improve thermal conductivity and/or energy storage capacity [33,34,35]. There are extensive reviews that have discussed the role of nanoparticles in enhancing the performance of phase change materials and molten salts, spanning fundamental research, synthesis, characterization, and applications [32,33,34,35]. Recent advancements in nanotechnology have further extended the design possibilities for advanced TCMs [36,37,38,39]. Carbon-based nanomaterials like graphene and carbon nanotubes (CNTs) have demonstrated exceptional capabilities in enhancing heat transfer (with thermal conductivity exceeding 3000 W/m·K [40,41,42]) and reaction kinetics in TES systems [43,44,45,46,47]. Similarly, incorporating metal oxide nanoparticles (e.g., SiO_2_, Al_2_O_3_, TiO_2_) into reversible reaction-based materials has proven to be effective in mitigating sintering and improving cycling stability [48,49]. Innovative approaches, such as atomic-scale doping and 3D-ordered macroporous structures [50], have led to significant improvements in energy storage density and reaction reversibility [51,52]. These nano-engineered composites not only overcome the intrinsic limitations of pure TCMs but also enable new functionalities through tailored porous nanostructures. Such breakthroughs highlight the transformative potential of nanotechnology in advancing TCES systems toward practical applications.

However, the successful incorporation of nanoparticles into TCMs requires a comprehensive understanding of how nanoengineering influences the thermal performance across different TCM classes. Most prior works either concentrate on a single thermochemical material system (e.g., such as CaO/Ca(OH)_2_), where nanoparticle effects are briefly mentioned, or lack in-depth insights into the underlying mechanisms of the nanomaterial–TCM interactions, including the morphology-performance relationships, enhanced contact by porous matrices, functional groups induced hydrogen-bonding, and dopant-driven inhibition of sintering, which have not been comprehensively analyzed in previous work. To this end, this review for the first time provides a systematic analysis of the latest progress in nano-engineered composite TCMs, addressing both fundamental challenges and innovative solutions. By systematically examining both sorption-based and reversible reaction-based systems, we aim to illustrate the critical role of nanotechnology in overcoming the existing limitations of TCES materials. Furthermore, we discuss remaining challenges and future research directions, offering insights into pathways for commercialization and widespread adoption.

## 2. Scope and Guidance

**Figure 2:**
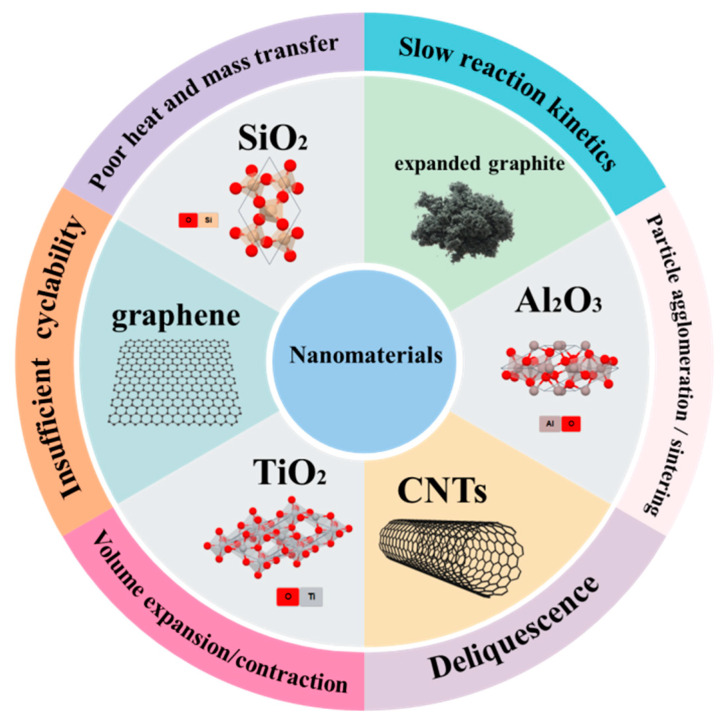
Scope and the key focus of this review.

This work aims to provide a comprehensive review on the latest advancement of the nano-engineered TCM composite, with the focus to reveal how nanoengineering addresses the challenges of different TCM classes, including agglomeration, deliquescence, poor cyclability, slow reaction kinetics, low thermal conductivity, poor heat, and mass transfer (see Figure 2). The literature compilation for this review was conducted through a systematic search of major scientific databases, including Web of Science, Scopus, and Google Scholar, covering publications from 2004 to 2024 to capture the most relevant advancements. The search is based on keywords such as “thermochemical energy storage”, “nanomaterials”, “nanoparticles”, “TCM”, “sorption”, “redox”, etc. The selection of the literature is limited to nanosized TCMs and components, either reactants, dopants, or matrices. In addition, works in the literature selection that were focused solely on thermal energy storage applications, thermochemical fuel production (e.g., H_2_, CO), or chemical synthesis were explicitly excluded. Furthermore, studies focusing on sensible or latent heat storage were also excluded to maintain a sharp focus on the review’s core subject.

This review begins with an analysis of intrinsic limitations in conventional TCMs, then the discussion transitions to cutting-edge nano-structuring strategies such as nanoparticle doping, porous nano-matrices, and flow-improving nano-agents. The review comprehensively evaluates how these nano-engineered approaches enhance reaction kinetics, thermal conductivity, and long-term stability, covering both sorption and reversible reaction-based TCMs. Ultimately, this review offers researchers and engineers a balanced perspective on the current state and future potential of nano-engineered TCMs in thermal energy storage (TES) systems. 

## 3. Overview of Thermochemical Heat Storage Materials

**Figure 3:**
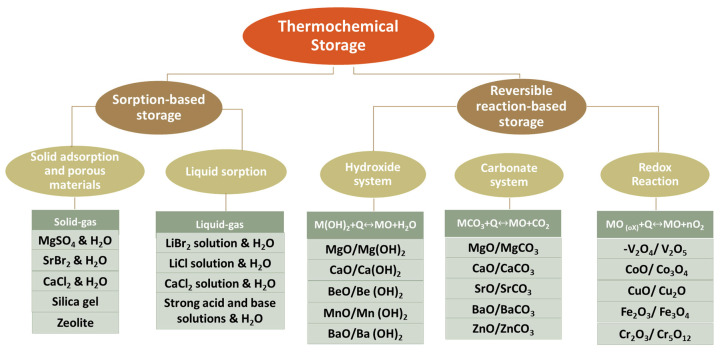
A broad classification of thermochemical materials.

Thermochemical energy storage systems can be classified into two main categories based on their working mechanisms: sorption-based systems and reversible reaction-based systems; see Figure 3. Sorption-based TCMs include physical sorption and chemical sorption materials, offering fast kinetics, and are suitable for low-to-medium temperature applications, e.g., domestic heating. Reversible reaction-based systems convert heat energy into chemical potential energy during a reversible reaction, which possesses a higher energy storage density and a wider operating temperature range compared to sorption systems [53]. In this work, we focus on the most advanced and potentially attractive TCES systems, including sorption TCES systems (e.g., salt hydrates) and reversible reaction TCES systems (e.g., hydroxides, carbonates, metals oxides redox pairs), with an emphasis on their characteristics for practical implementation.

### 3.1. Sorption Thermochemical Heat Storage Materials

Sorption TCMs target low-grade heat sources (below 250 °C) and are well-suited for building applications [18,54]. Sorption refers to a fixation or capture process of a gas or vapour, which includes adsorption and absorption process, with the former being a surface phenomenon and the latter a volume phenomenon [55]. Depending on the interactions between sorbate and sorbent, the sorption can be classified into either or both thermophysical (physisorption, mainly due to the Van der Waals forces) and thermochemical processes (chemisorption, mainly due to chemical bonds). In terms of the physical state of the sorbent, there are liquid sorbents and solid sorbents. Table 1 provides a comprehensive comparison between different types of sorption TCMs.

#### 3.1.1. Liquid Sorption

Liquid sorption is a chemical/physical phenomenon that occurs when liquid sorbent penetrates into the surface layer of a sorbent, entering the structure of the sorbent, and results in the changing of its composition [55,57]. For TES application, hygroscopic inorganic salt solutions are commonly used as a liquid absorption working pair. Strong acids, such as H_2_SO_4_ are also considered as alternatives for absorption heat pumps or energy storage systems, however they suffer from a corrosive nature, safety hazards, and material compatibility issues. Lahmidi and Mauran [58] conducted experimental studies on the SrBr_2_/H_2_O sorption working pair, demonstrating its potential for residential heating applications. It had a regeneration temperature below 80 °C, making it particularly suitable for integration with conventional flat-plate solar collectors. A lab-scale prototype thermochemical heat storage system based on the SrBr_2_/expanded graphite composite also shows a remarkable storage capacity over 250 kWh/m^3^ with the prototype size of 60 kWh for heating and 40 kWh for cooling [59]. The LiCl/H_2_O working pair has impressive storage capacities: a cold storage density of 519 kWh/m^3^ when charged at 90 °C in summer conditions, and a heat storage density of 618 kWh/m^3^ (1250 Wh/kg) when charged at 75 °C in winter conditions [60]. Many kinds of absorption materials have been considered in energy storage systems, but their prototypes suffer from severe corrosion issues during practical application [61,62].

#### 3.1.2. Solid Sorption

**Figure 4:**
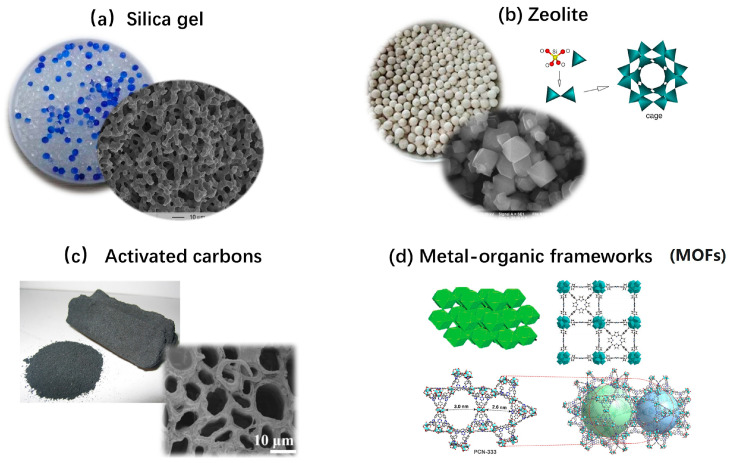
Typical physical adsorbents [82], “reprinted with permission from Ref. [82]. Copyright (2025), Wiley”.

Solid sorbent materials are categorized into physical and chemical sorbents based on their interactions with sorbates. Physical sorbents (see Figure 4), such as zeolites and silica gels, rely on Van der Waals forces for adsorption, while chemical sorbents (e.g., salt hydrates) form chemical bonds through reversible hydration/dehydration reactions.

Silica gel stands as one of the most prevalent physical adsorbents, given its cost-effectiveness and moderate regeneration temperatures (130–150 °C) [16]. Its hydrophilic nature and high specific surface area (750–850 m^2^/g [63]) also facilitate efficient water molecule adsorption, yielding substantial adsorption heat outputs of approximately 2712 kJ/kg [64]. However, the material’s structural integrity presents significant challenges, as it demonstrates a tendency to undergo structural deformation during repeated hydration/dehydration cycles, particularly when exposed to saline solutions, ultimately resulting in powder formation and compromised durability [65]. Zeolites, particularly types 13X, 10X, 4A and 5A, are also regarded as promising TCMs [66,67,68,69], with zeolite 13X demonstrating the highest adsorption capacity [70]. These crystalline aluminosilicates combine exceptional hydrophilicity with well-defined pore structures, offering superior water adsorption capabilities compared to silica gels. Their stronger water molecule interactions also make them attractive candidates, though the high regeneration temperatures exceeding 200 °C present practical limitations. Metal–organic frameworks (MOFs) represent a cutting-edge class of adsorbent materials characterized by exceptional porosity [71], with specific surface areas exceeding 6000 m^2^/g and large pore volumes [72]. These materials feature precisely engineered 3D networks of metal clusters connected by organic linkers, creating tuneable pore architectures that are ideal for adsorption applications. In addition to the aforementioned adsorbents, researchers have explored alternative materials for water vapour adsorption, including activated alumina (Al_2_O_3_) [73], clay minerals [74,75], and activated carbons [76]. In addition, a particularly noteworthy approach involves utilizing these porous materials as host matrices to provide the composite structure, thereby synergistically combining their excellent vapour adsorption properties with good mechanical stability [77,78,79,80,81].

Chemical adsorbents (e.g., salt hydrates) show higher energy density through strong chemical bonding, while physical adsorption enables faster kinetics and better cycling stability. The generic adsorption–desorption process can be expressed by Equation (1), where the adsorption–desorption process in these materials usually follows a stepwise hydration/dehydration mechanism.

(1) $$Salt\cdot xH_{2}O+∆H↔Salt+xH_{2}O$$

Table 2 listed the characteristics of selected sorption materials. Despite their high theoretical energy density, practical application of pure salt hydrates faces significant challenges. For example, MgSO_4_·7H_2_O suffers from slow reaction kinetics, while LiCl and CaCl_2_ exhibit deliquescence behaviour under high RH conditions. These limitations necessitate the development of advanced composite materials through several approaches: (1) impregnation of salts into porous matrices to enhance stability and prevent agglomeration, (2) polymeric stabilization to improve mechanical integrity, and (3) the formulation of binary/ternary salt mixtures to optimize overall performance.

### 3.2. Reversible Reaction-Based Thermochemical Heat Storage Materials

Reversible reaction-based thermochemical heat storage utilizes chemical bonds to store and release heat, possessing high energy density and minimal thermal losses over time. The energy charging process is realized by the dissociation of reactant AB into the products A and B and stored separately. There are various reaction types; here, we focus on the most investigated reactions below: (1) hydration/dehydration reactions, where water vapour reversibly binds to metal oxides; (2) carbonation/decarbonation, exploiting reversible CO_2_ capture; (3) redox reactions, involving oxygen exchange in metal oxides. Each class offers distinct temperature ranges and applications, with trade-offs in cyclability, kinetics, and material stability.

#### 3.2.1. Hydroxide System

Metal hydroxides are able to conduct reversible dehydration/rehydration reactions at medium to high temperatures, usually 70 °C to 1000 °C [48]. The thermochemical reactions are as follows:

(2) $${M(OH)}_{2}+∆H↔MO+H_{2}O$$

Among diverse metal hydroxide systems, the Ca(OH)_2_/CaO system has drawn significant interest for energy storage applications due to its high enthalpy of reaction, fast kinetics and cost-effectiveness. Experimental studies have demonstrated its good reversibility and cycling stability. Schmidt et al. [85] conducted large-scale tests and achieved a 77% reversible conversion of Ca(OH)_2_. At the laboratory scale, Schaube et al. [86] investigated the thermodynamics and reaction kinetics of Ca(OH)_2_, observing stable cycling over 100 cycles. Their results showed complete conversion with a reaction enthalpy of 104.4 kJ/mol at an equilibrium temperature of 505 °C under 1 bar of H_2_O partial pressure. MgO/Mg(OH)_2_ is employed to store thermal energy at temperatures higher than 300 °C [87]. Magnesium hydroxide also presents a high enthalpy of reaction [87]. However, the material suffers from slow and incomplete rehydration, as stated by Müller et al. [88], who recently studied the rehydration mechanism of MgO and natural magnesite to assess the effect of impurities on the reaction.

#### 3.2.2. Carbonate System

Metal carbonates present the advantage of being cheap and largely available materials, and they have emerged as favourable reactants due to their ability for CO_2_ capture [89,90,91]. The general carbonation of metal oxides for TCES occurs at high temperatures (120 °C to ~1500 °C) and has the following form:

(3) $${MCO}_{3}+∆H↔MO+CO_{2}$$

There are several metal carbonates that show attractive performances for TCES application, such as CaO/CaCO_3_, SrO/SrCO_3_ or BaO/BaCO_3_ [89,90,91]. The CaCO_3_/CaO system has emerged as a particularly promising candidate for integration in concentrating solar power (CSP) plants, operating effectively within the 850–950 °C temperature range while offering a substantial reaction enthalpy of 1656.8 kJ/kg. However, this system faces significant challenges related to material sintering, which progressively diminishes porosity and limits reactive gas access to active sites [92,93]. Recent research efforts have consequently focused on enhancing the cyclic performance of CaO-based sorbents through material modifications. MgCO_3_ also demonstrates notable CO_2_ capture capabilities at lower operating temperatures [94,95], while SrCO_3_ has shown cycling degradation issues, particularly sintering at 1200 °C [96]. Recent studies on BaO/BaCO_3_ also reveal significant sintering-induced degradation at operating temperatures above 1200 °C [97].

#### 3.2.3. Redox System

Reduction-oxidation (Redox) reactions generally occur at temperatures between 400 °C and 1700 °C and have the following form:

(4) $${MO}_{x}+∆H↔MO_{x\;-\;y}+{\frac{x-y}{2}O}_{2}$$

Metal oxides offer distinct advantages due to their compatibility with air as a heat transfer fluid, enabling open-loop operation. This contrasts with metal sulphates or carbonates that require closed-loop systems with specialized fluids (SO_2_/CO_2_), or hydroxides that need steam generation. Wu et al. [98] systematically screened various pure and the mixed metal oxide redox systems for high-temperature thermochemical energy storage applications, and identified Co_3_O_4_/CoO as the best-performing metal oxide due to its exceptional energy density and reversibility. However, its commercial viability is limited by cost and toxicity concerns. Neises et al. [99] tested cobalt oxide in solar-heated rotary kilns (900 °C, air atmosphere), which maintains ~400 kJ/kg storage capacity over 30 cycles, though incomplete reduction (~50% conversion) occurred due to mixing limitations. Alternative systems including Mn_2_O_3_/Mn_3_O_4_, CuO/Cu_2_O and Fe_2_O_3_/Fe_3_O_4_ present more economical and environmentally advantages, which are therefore becoming the focus for high-temperature TCES applications.

## 4. Fundamental Challenges of Pure Thermochemical Materials

Pure thermochemical heat storage materials encounter multiple scientific and technical challenges that hinder their practical implementation. The major challenges include the following:

- The slow reaction kinetics and poor heat and mass transfer of the pure TCMs, which are influenced by temperature, pressure, and material properties (e.g., intrinsic thermal conductivity, pore structure, thermal expansion [100]), restricting energy output and system efficiency.
- Insufficient cyclability is another critical issue, while commercial applications demand 3000–5000 cycles, most lab-tested materials fail after merely 20–100 cycles due to cumulative structural and chemical degradation.
- Particle agglomeration or sintering occurs over repeated cycles due to increased interparticle bonding, further decelerating mass transfer and long-term stability.
- Volume expansion/contraction during hydration–dehydration cycles is also an issue which induces mechanical stress, leading to structural fractures and performance degradation.
- Deliquescence presents an issue, especially for salt hydrate-based sorption TCMs, where hygroscopic salts absorb water vapour beyond intended levels, disrupting reaction equilibrium.

Researchers are pursuing material-level innovations, such as composite formulations with inert binders to buffer volume changes and prevent agglomeration and sintering, and the incorporation of porous matrices and high-conductivity additives to enhance kinetics and heat transfer. Other than those, recent advances in nanotechnology offer promising strategies to overcome these limitations of conventional TCMs.

## 5. Nano-Engineered Composite Thermochemical Materials

### 5.1. Sorption-Based TCMs

Sorption-based thermochemical heat storage materials, particularly salt hydrates, have been extensively investigated for medium temperature thermal energy storage. However, inherent limitations of pure inorganic salts, including slow hydration kinetics and low thermal conductivity, restrict their practical implementation. Hence, the preparation of heat storage composite TCMs with strong water sorption and high thermal conductivity is of great importance.

**Figure 5:**
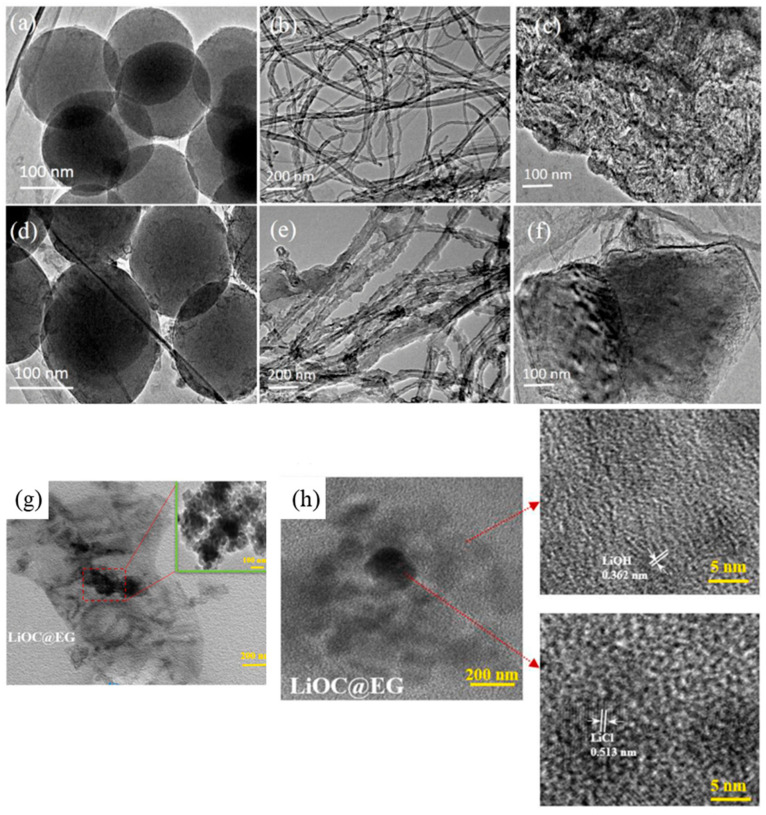
TEM images of (**a**) CNSs, (**b**) MWCNTs, (**c**) LiOH·H_2_O, (**d**) LiOH·H_2_O/CNSs, (**e**) LiOH·H_2_O/MWCNTs, (**f**) LiOH·H_2_O/AC [43], and (**g**,**h**) LiOH/LiCl/expanded graphite [106], “reprinted with permission from Ref. [106]. Copyright (2025), Elsevier”.

Carbon-based nanomaterials, including carbon nanotubes (CNTs) [44,45] and carbon nanospheres (CNSs) [101], activated carbon (AC), porous biochar [102], expanded graphite (EG) [103], and graphene [50], have emerged as effective functional nano-additives due to their exceptional specific surface area (typically 100–1000 m^2^/g), high thermal conductivity (>3000 W/m·K for single-walled CNTs), and chemical stability. Yang et al. [43] incorporated Carbon nanospheres (CNSs) and multi-walled carbon nanotubes (MWCNTs) into lithium hydroxide monohydrate, finding that these nano-additives dispersed LiOH·H_2_O into nanoscale particles (20–100 nm), as seen in Figure 5. The resulting composites demonstrate significantly improved heat storage densities, reaching 2020 kJ/kg for CNS-modified and 1804 kJ/kg for MWCNT-modified systems, representing a 3–5-fold enhancement compared to pure LiOH·H_2_O (661 kJ/kg). The enhanced hydration kinetics stem from two synergistic effects: (1) the high specific surface area of carbon nanomaterials enables uniform LiOH·H_2_O nanoparticle dispersion, maximizing reactive interfaces; and (2) surface hydrophilic oxygen-containing functional groups (hydroxyl/carbonyl groups) on the surface of the CNSs, MWCNTs, and AC facilitate water adsorption and create favourable hydrogen-bonding networks at the reaction interfaces. Advanced 3D architectures, such as nickel-CNT (Ni-CNT) networks, were prepared by a catalytic chemical vapour deposition method and incorporated with LiOH·H_2_O [104]. The authors found the nanostructure can further optimize the performance of LiOH·H_2_O by reducing activation energies and providing efficient hydrophilic interfaces. Hydrogen bonding formed between H_2_O and hydrophilic groups on the surface of Ni-CNTs leads to the improved hydration rate [105]. As a result, these systems achieve remarkable storage capacities up to 3935 kJ/kg, with the energy density is 5.9 times higher than that of pure lithium hydroxide. The 3D nanostructured nickel-CNT also enhanced the heat conduction by introducing highly conductive percolation networks, thus enhancing the thermal conductivity from 1.69 to 3.78 W/m·K. Expanded graphite (EG) matrices have also proven to be effective, enabling hierarchical micro-nano structures for relatively high salt loadings. Li et al. [106] developed expanded graphite (EG)-based composites containing hierarchical micro-nano structured LiOH/LiCl particles with salt loadings exceeding 60 wt%. Microscopic analysis revealed a distinctive core–shell architecture, where smaller LiOH crystallites (20–50 nm) nucleated around larger LiCl crystals (100–200 nm) within the EG matrix; see Figure 5h. These composites demonstrated exceptional energy storage performance, achieving volumetric energy storage densities surpassing 200 kWh/m^3^. Notably, the materials exhibited outstanding cycling stability, retaining 95–96% of their initial capacity after 20 dehydration–hydration cycles, confirming their structural integrity and practical applicability for thermal energy storage systems. Ousaleh et al. [107] utilized natural bentonite combined with graphite (BNTC) as a supporting material to stabilize three hydrated salts: SrCl_2_⋅6H_2_O, CaCl_2_⋅6H_2_O, and LiCl⋅H_2_O. The composite exhibited both high thermal conductivity and significant energy storage density. Cyclability tests revealed that over 90% of the stored heat was retained after 10 cycles, confirming the effectiveness of BNTC in addressing the deliquesces challenge of hydrated salts. These advancements demonstrate the critical role of carbon-based nanomaterial engineering in developing high-performance sorption TES systems with optimized kinetics and stability.

### 5.2. Reversible Reaction-Based TCMs

#### 5.2.1. Hydroxide System

**Figure 6:**
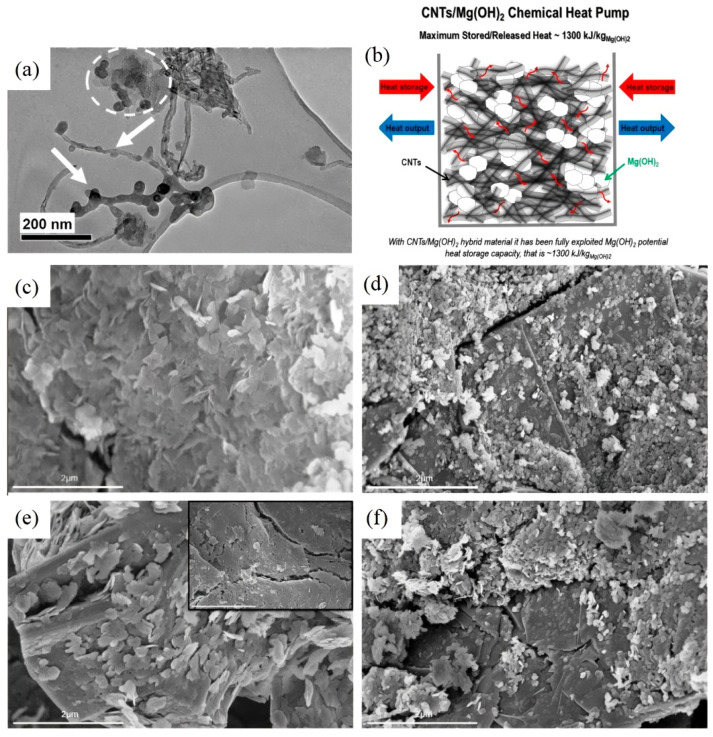
TEM images of (**a**,**b**) Mg(OH)_2_/CNTs [45], “reprinted with permission from Ref. [45]. Copyright (2025), Elsevier”. (**c**–**f**) SEM images of Mg(OH)_2_/graphite prepared via deposition–precipitation synthesis method [44], “reprinted with permission from Ref. [44]. Copyright (2025), Elsevier”.

Metal hydroxide systems, operating in the range of 250–500 °C, present promising candidates for medium-high temperature heat storage. Expanded graphite (EG) serves as an effective thermal conductivity enhancer and reaction promoter to incorporate with Mg(OH)_2_; the modified systems demonstrated a significant improvement in both heat storage capacity (reaching ~1300 kJ/kg) and thermal output rates compared to pure Mg(OH)_2_ [103,108]. This enhancement stems from EG’s dual role as both a thermal conduction network and a nanostructural template, which simultaneously improves heat transfer while maintaining the active material’s dispersion. Nano-porous carbon (NC), characterized by its well-defined mesoporous structure and narrow pore size distribution, was employed by Deng et al. [101]. The NC-based composites demonstrated a significant improvement in energy storage capacity, achieving a heat storage density of 1053 kJ/kg. This outperforms other comparable systems by more than 30%, owing to NC’s optimized porous architecture. However, directly incorporating Mg(OH)_2_ with carbonaceous additives may lead to weak interaction, which may cause poor durability during repeated cycles. To address the poor interfacial compatibility between inorganic Mg(OH)_2_ and organic carbonaceous materials in conventional composites, researchers have developed an innovative deposition–precipitation synthesis method [44,45]. This approach enables the direct growth of Mg(OH)_2_ nanocrystals on exfoliated graphite surfaces in alkaline aqueous media (pH ~ 11.5), where electrostatic interactions between negatively charged graphite and positively charged Mg(OH)_2_ surfaces ensure optimal interfacial bonding [44]. The enhanced interfacial contact led to a thin layer of Mg(OH)_2_ nanoparticles being finely dispersed on the CNT/EG surface (see Figure 6), yielding a great heat storage capacity up to 1200 kJ/kg. Furthermore, better interfacial compatibility and dispersibility can be achieved by the insertion of oxygenated functionalities on the carbonaceous material’s surface [45]. The surface modification enabled superior nanoparticle dispersion within CNT networks (see Figure 6), ultimately achieving a record heat storage capacity of 1300 kJ/kg.

**Figure 7:**
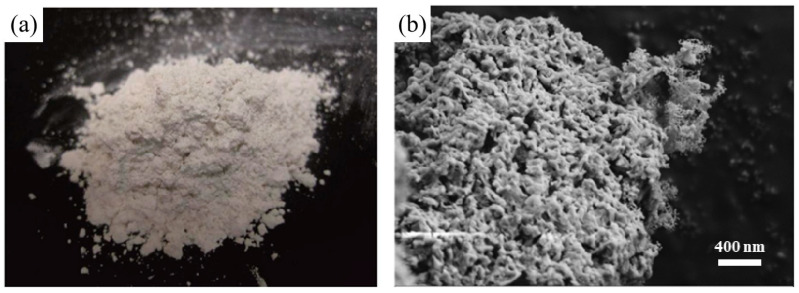
Ca(OH)_2_ mixed with SiO_2_ after four cycles in the lab scale reactor: (**a**) sample picture; (**b**) SEM images [109]. These were “reprinted with permission from Ref. [109]. Copyright (2025), Elsevier”.

**Figure 8:**
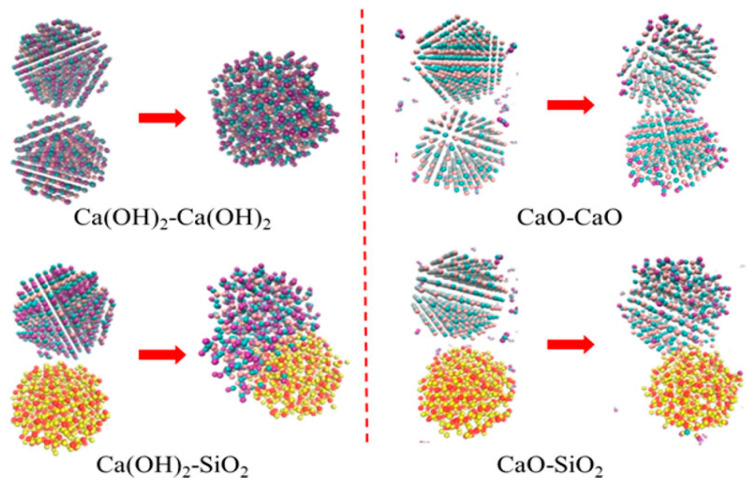
The impact of nano-SiO_2_ dopants on agglomeration behaviour of Ca(OH)_2_ [110], “reprinted with permission from Ref. [110]. Copyright (2025), American Chemical Society”.

To prevent the agglomeration and sintering of the metal hydroxide system, Roßkopf et al. [109] first introduced SiO_2_ nanoparticles as a coating material for Ca(OH)_2_/CaO. The introduction of SiO_2_ nanoparticles minimized the interparticle attractive forces and the associated particle cohesion, leading to stabilized thermophysical bulk properties (see Figure 7). Xu et al. [110] used molecular dynamics (MD) simulations to analyze the impact of inert dopants on agglomeration behaviour. Reactive force field simulations revealed that H_2_O significantly accelerates the agglomeration of Ca(OH)_2_ due to enhanced atomic displacements during thermochemical reactions. However, incorporating SiO_2_ nanoparticles helps reduce agglomeration by creating physical gaps between reactant particles, weakening cohesive forces (see Figure 8). Pardo et al. [48] doped Ca(OH)_2_ powder with Al_2_O_3_ powder, reaching promising energy densities. Jashari et al. [49] also investigated different nanostructured coatings (Al_2_O_3_, SiO_2_, and Al_2_O_3_/SiO_2_ mixtures) on Ca(OH)_2_ granules. The study found that Al_2_O_3_-coated granules, post-treated at 600 °C, exhibited the best balance of cycling stability, structural integrity, and mechanical strength, maintaining a consistent 88% conversion rate over multiple cycles. However, a key challenge with nanoparticle additives in a CaO/Ca(OH)_2_ system is the potential for side reactions. For instance, SiO_2_ and Al_2_O_3_ can react with Ca(OH)_2_, forming compounds like calcium silicates [111] and calcium aluminates [49], and reinhardbraunsite Ca_5_(SiO_4_)_2_(OH)_2_ [109]. These various side products have different effects on the stability and persistence of the granules. These byproducts may influence granule stability, reactivity, and long-term performance.

**Figure 9:**
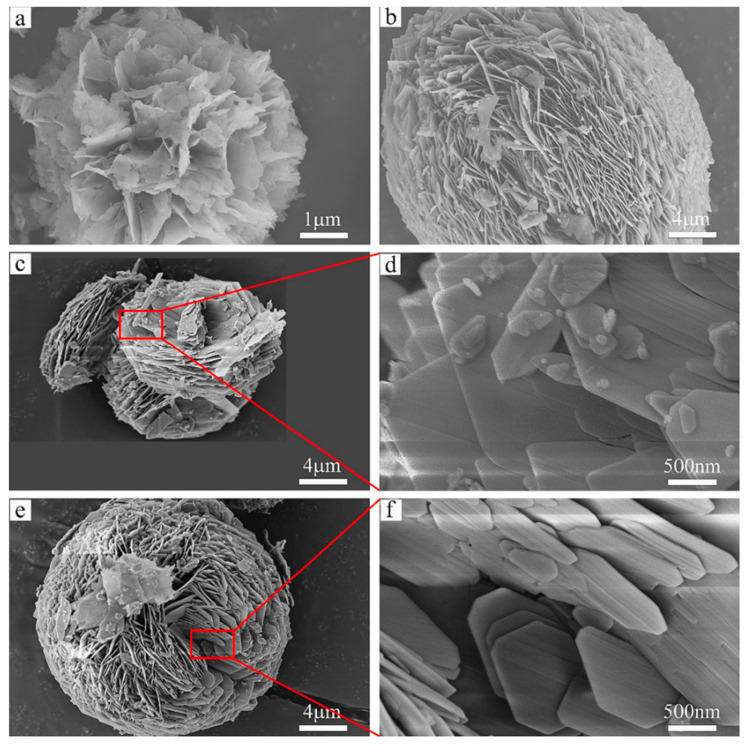
SEM images of self-assembled micro-nano flower-like magnesium (**a**) and spherical magnesium by microwave-assisted hydrothermal synthesis for 45 min (**b**), 15 min (**c**,**d**) and 30 min (**e**,**f**) [51], “reprinted with permission from Ref. [51]. Copyright (2025), Elsevier”.

Increasing the reactant’s specific surface area is an effective approach to improve hydration performance; therefore, some authors synthesized nanosized metal hydroxides with a high specific surface area for practical thermochemical heat storage applications. Han et al. [51] developed a large-scale synthesis approach for fabricating self-assembled micro-nano flower-like and spherical magnesium oxide structures using a microwave hydrothermal method. As shown in Figure 9, these hierarchical structures exhibit remarkably high specific surface areas of 255.1 m^2^/g (flower-like) and 181.5 m^2^/g (spherical), respectively. The materials demonstrate superior thermal performance with effective exothermic densities reaching 955.6 kJ/kg and 1034.8 kJ/kg, which is approximately 2–3 times higher than those of non-treated magnesium hydroxide. Additionally, their average pre-hydration rates are 6–7 times greater than those of Mg(OH)_2_, indicating that the introduction of porous structures effectively addresses the slow and incomplete hydration issues of magnesium oxide. Kim et al. [52] employed an alternative approach by creating micro-beam structures on MgO pellet surfaces through electron beam irradiation. This method enabled the large-scale production of Mg(OH)_2_ flakes via water reaction on the enhanced MgO surface area, without requiring dispersants or generating a fine powder. The technique shows promising potential for application in various thermal energy storage systems due to its scalability and suitability for large-area processing. Huang et al. [112] synthesized Ca(OH) _2_ nanoparticles with spindle-like and hexagonal morphologies using a deposition-precipitation method. BET analysis revealed that the spindle-shaped Ca(OH)_2_ exhibited the highest specific surface area and pore volume among the samples, corresponding to superior heat storage and output capacities of approximately 1300 kJ/kg. Furthermore, the spindle-shaped particles demonstrated the shortest dehydration/hydration time, suggesting that the morphology of calcium hydroxide particles directly influences the reaction kinetics. Even after ten dehydration/hydration cycles, the spindle-shaped Ca(OH)_2_ maintained a conversion rate above 70%.

**Figure 10:**
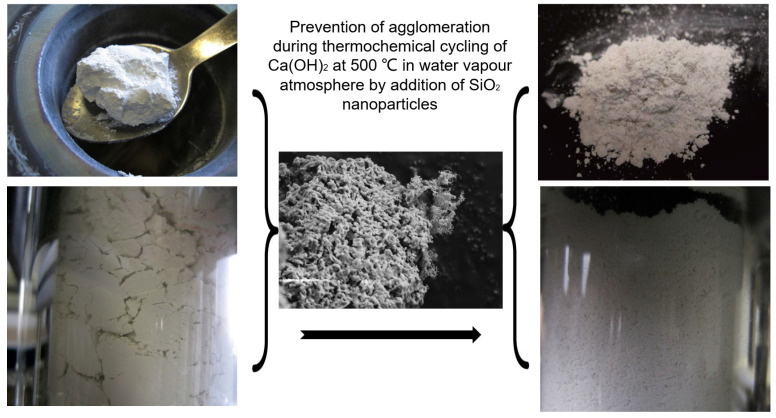
SiO_2_ nanoparticles serve as flow agents in a CaO/Ca(OH)_2_ system [109]. These images were “reprinted with permission from Ref. [109]. Copyright (2025), Elsevier”.

Nanoparticles also serve a dual purpose in thermochemical energy storage systems, functioning not only as dopants but also as agents to enhance the fluidization characteristics of bulk hydroxide materials. In this application, nanoparticle agglomerates act as host particles, while hydroxide particles adhere to their surfaces as guest particles. While this approach improves fluidization, it necessitates nanoparticle loadings as high as 30 wt%, which presents significant drawbacks for practical thermochemical storage applications due to substantial reductions in energy storage density and increased material costs. Roßkopf et al. [109,113] conducted pioneering investigations into the effects of silica nanoparticles in fluidized-bed reactors (see Figure 10), demonstrating improved fluidization while simultaneously identifying undesirable side reactions during thermal cycling. Gollsch et al. [114] systematically evaluated five different nanostructured fumed silica and alumina additives as flow-enhancing agents for Ca(OH)_2_ powders. Although initial assessments showed improvements in flowability for four of the additives, all modified powders still exhibited cohesive behaviour according to standardized flowability measurements. Subsequent thermochemical cycling revealed a significant degradation in flowability across all nanoparticle-modified samples, contrasting with results obtained for silica-modified limestone powders used in CO_2_ capture applications [115]. This paradoxical behaviour can be attributed to the formation of interfacial reaction products between the nanostructured additives and host CaO/Ca(OH)_2_ particles. These side reactions appear to modify particle surface morphology, leading to increased interparticle forces as surfaces become smoother.

#### 5.2.2. Carbonate System

**Figure 11:**
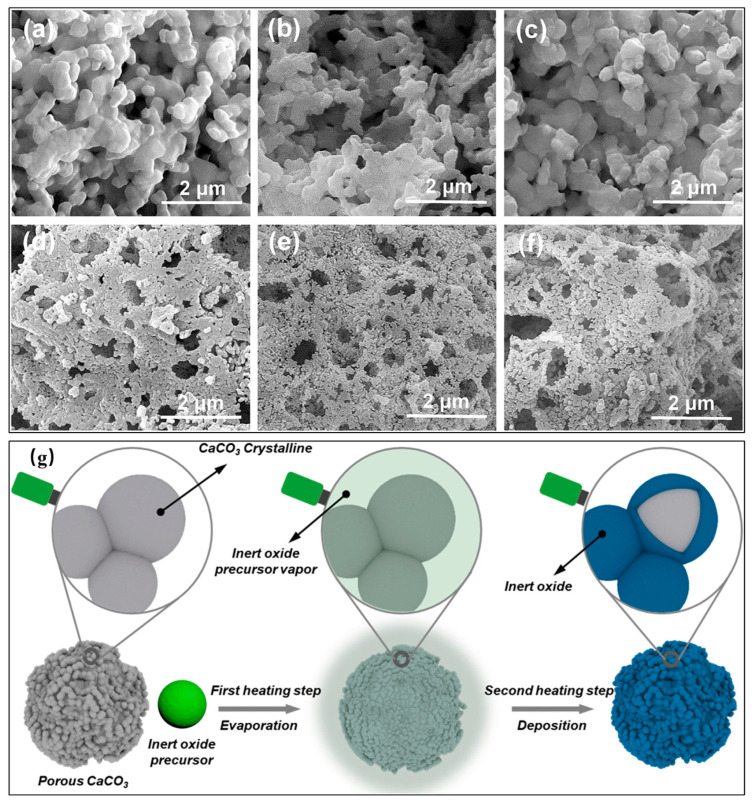
SEM images of CaO coated with Al_2_O_3_, SiO_2_, or TiO_2_: fresh (**a**–**c**), calcined (**d**–**f**), and (**g**) the formation mechanism by chemical vapour deposition method [125]. These images were “reprinted with permission from Ref. [125]. Copyright (2025), Elsevier”.

The CaCO_3_/CaO thermochemical system faces challenges including incomplete reaction kinetics and slow heat storage rates. To enhance reaction reversibility, two key strategies have been identified: (1) improving CO_2_ diffusion through the material matrix, and (2) mitigating CaO particle sintering. To optimize system performance, decarbonation should be carefully controlled below 925 °C to minimize the sintering of the newly generated CaO [116]. Furthermore, incorporating inert stabilizing materials has proven to be effective for enhancing the high-temperature cyclic stability, for instance, SiO_2_, MgO [117,118], Mn [119], CeO_2_ [117,118], and TiO_2_ [120]. Khosa et al. [116] found that incorporating SiO_2_ nanoparticles (15 nm) at a 1:1 molar ratio improved reaction kinetics at 700 °C while lowering the decarbonation temperature and enhancing cyclic stability by 13%. Similarly, valverde et al. [121] incorporated nano-SiO_2_ to improve the dispersibility of CaO agglomerates, which contributes to a larger number of small pores and the associated enhanced contact area, thus improving the thermal stability and performance. However, a high proportion of SiO_2_ (15% and 30%) could lead to the generation of a new phase of Ca_3_SiO_5_ with CaO materials [122]. Han et al. [123] developed CaCO_3_/graphite nanosheet composites through a one-pot synthesis method, showing that just 3 wt% graphite loading could dramatically boost the system’s performance. These composites achieved a remarkable heat storage capacity of 1313 kJ/kg after 50 cycles, which is 2.9 times that of pure CaCO_3_. The graphite nanosheet was found to prevent particle sintering through the uniform dispersion CaCO_3_ particles on its surface. Other studies have explored oxide additives like nano-Al_2_O_3_ [124], which observed significant microstructural modifications. The nano-Al_2_O_3_ integration yielded a substantially higher fraction of grains smaller than 500 nm compared to untreated samples. The authors also observed that the addition of nano-Al_2_O_3_ could initially reduce conversion due to calcium aluminate formation, but ultimately improved long-term stability under Ca-looping (CaL) in a concentrated solar power plant (CSP). Simple synthesis methods, such as ball-milling and co-precipitation, are hard to use to realize the nanoscale mixing of CaO and inert oxide stabilizers. Hence, Han et al. [125] developed a space-confined chemical vapour deposition (CVD) method to fabricate highly efficient CaO-based thermal storage materials. In the process, Al_2_O_3_, SiO_2_, or TiO_2_ were uniformly deposited onto CaCO_3_ grains (see Figure 11), with Al_2_O_3_ proving to be the most effective stabilizer. A composite with only 5 mol.% Al exhibited exceptional cyclic stability, achieving an energy density of 1.50 GJ/t after 50 cycles without degradation. Jiménez et al. [126] also utilized acicular calcium and magnesium acetate precursors, which decompose to form an open carbonate structure with remarkable resistance to sintering. This structure effectively maintains high CaO reactivity over numerous cycles, through a stable porous structure comprising well-dispersed MgO nanoparticles coating the CaO/CaCO_3_ grains. This morphology not only resists pore-plugging and sintering but also ensures high long-term effective conversion.

**Figure 12:**
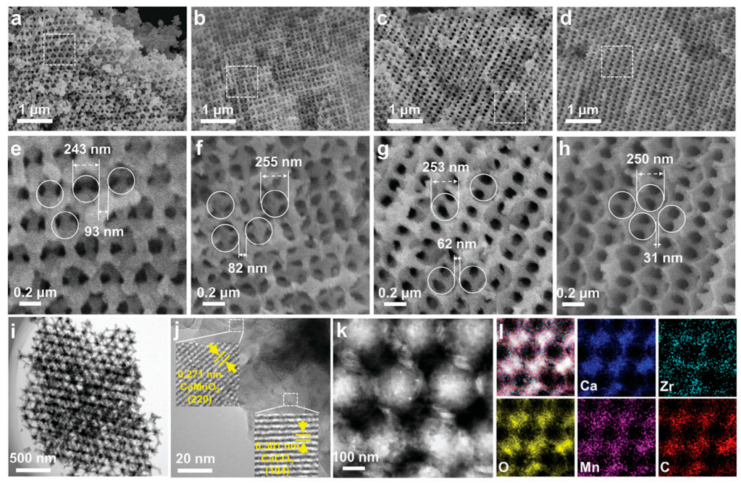
SEM images (**a**–**h**), TEM and HRTEM images (**i**,**j**), HAADF-STEM and EDX elemental mapping images (**k**,**l**) of as synthesized 3DOM Ca-based samples [127].

More sophisticated approaches involved the development of a series of Mn and Zr co-doped CaCO_3_ nanomaterials with 3D ordered macroporous (3DOM) structure via a templated co-precipitation method (see Figure 12) [127]. The hierarchically mesoporous/macroporous structure yielded exceptional results including an ultra-high initial storage density (1706.4 kJ/kg), excellent solar absorptance (74.1%), and minimal capacity loss (<6%) after 125 cycles. These materials benefit from both the atomic dispersion of dopants in the CaCO_3_ lattice and the formation of stabilizing CaMnO_3_ nanoparticles. As indicated by the density functional theory (DFT) calculations, the co-incorporation of Mn and Zr into the crystal lattice of CaO offered a strong interfacial affinity to prevent CaO nanoparticles from sintering and agglomeration. Bian et al. [128] also developed a hollow nanostructured CaO-based material via a carbonaceous templating method, using calcined limestone as a low-cost calcium precursor for applications in CO_2_ capture and CaO/Ca(OH)_2_ heat storage. The resulting hollow nanostructure (HN-CaO) exhibits a specific surface area that is 1.5 times larger than that of conventional calcined limestone. This hierarchical architecture facilitates the diffusion of CO_2_ and steam both between nanoshells and across inner and outer surfaces, significantly reducing diffusion resistance. As a result, the HN-CaO achieves a CO_2_ capture capacity of 0.45 g/g after 20 cycles, which is 1.7 times higher than that of limestone-derived CaO. Furthermore, several heat storage cycles were found to improve the pore structure of the sintered hollow CaO, leading to a 94% enhancement in CO_2_ capture capacity in subsequent cycles.

#### 5.2.3. Redox System

**Figure 13:**
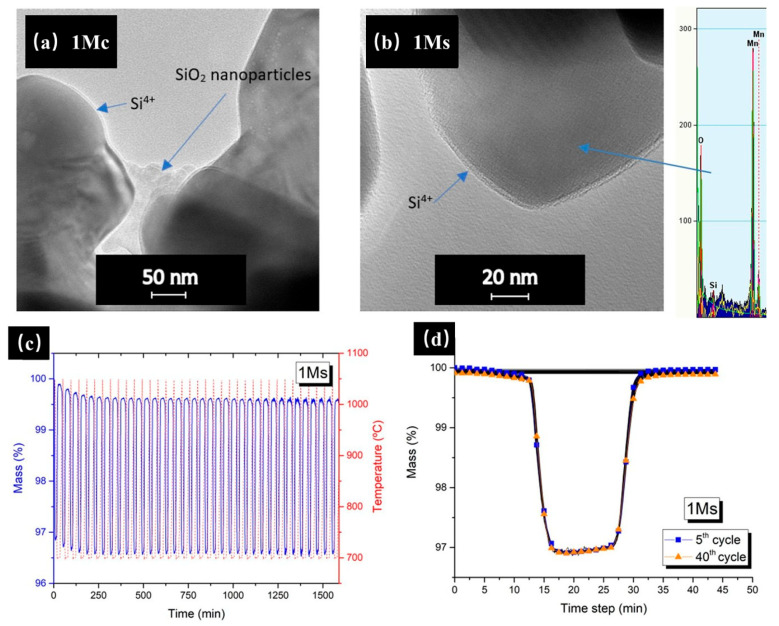
TEM images of Mn_2_O_3_ doped with 1% Si after cycling via (**a**) an dry mixing method (1Mc) and (**b**) a sol–gel method (1Ms); and (**c**,**d**) TGA analysis of sol–gel-prepared samples for 40 cycles [130]. These images were “reprinted with permission from Ref. [130]. Copyright (2025), Elsevier”.

Redox (reduction–oxidation) thermochemical storage offers a superior advantage through the ability to use air as both the reactant and the heat transfer medium, which enables open-loop design. Thermal cycling-induced sintering in cobalt-based oxides severely degrades material kinetics, causing capacity loss and an equilibrium shift. Recent studies have demonstrated significant advancements in enhancing the redox thermochemical storage performance through nano-material modifications. Portilla-Nieto et al. [129] explored the size effects of a SiO_2_ particle on the long-term thermochemical storage performance of Co_2.4_Ni_0.6_O_4_, finding that adding 400 nm SiO_2_ particles at 0.5 wt% significantly enhance its cyclability and reduce densification. Bielsa et al. [130] employed a sol–gel approach to fabricate Mn_2_O_3_/SiO_2_ nanocomposites, achieving the segregation of Si^4+^ cations along Mn_2_O_3_ grain boundaries (see Figure 13). This strategic dopant distribution demonstrated dual functions: (1) effectively suppressing grain coarsening and particle shrinkage through boundary pinning effects, while (2) simultaneously enhancing the material’s reoxidation kinetics. The synergistic combination of these mechanisms resulted in significantly improved cycling stability. Zhou et al. [131] developed a high-performance CuO-ZrO_2_ composite (30 wt% ZrO_2_) exhibiting exceptional redox characteristics, achieving a remarkable 99.7% reoxidation rate. The uniform dispersion of ZrO_2_ nanoparticles on CuO surfaces effectively suppresses particle growth through a unique interfacial stabilization mechanism. Molecular dynamics simulations revealed that ZrO_2_ incorporation elevates the atomic diffusion activation energy in CuO, fundamentally explaining the observed sintering resistance mechanism. These findings highlight the importance of particle size control, grain boundary engineering, and surface modification as effective strategies for enhancing cycling stability and maintaining storage capacity.

### 5.3. Comparison Between Different Nano-Engineered Thermochemical Materials

Table 3 shows the comprehensive comparison between different nano-engineered thermochemical materials. It is evident that nano-engineering strategies impart significant yet highly variable performance enhancements across different thermochemical material classes. Sorption-based systems, particularly those incorporating carbon nanomaterials like CNSs and 3D Ni-CNT networks, demonstrate the most dramatic improvements in energy density, achieving gains of 3–5.9 times over the pure materials (highest up to 3935 kJ/kg). This is primarily attributed to the exceptional specific surface area and surface hydrophilic oxygen-containing functional groups, which optimize water sorption kinetics. The extreme high intrinsic thermal conductivity of carbon additives also leads to a fast heat transfer rate. However, the high cost of the carbon nanomaterials hinders its large-scale applicability. For reversible reaction systems, the incorporation of nano-additives presents a critical trade-off between performance enhancement and side effects, and synthesis complexity. Hydroxide systems, for instance, benefit from improved hydration kinetics, sintering resistance, and cyclic stability through the use of additives such as expanded graphite (EG) and metal oxides (e.g., SiO_2_, Al_2_O_3_). However, these systems face challenges such as side reactions and the need for high nanoparticle loadings (up to 30 wt%) to act as flow-improving agents, which increases overall cost and complicates scalability. For carbonate systems, the side reaction between the TCM and the nanomaterials at high temperatures also presents as a significant issue. In addition, while advanced nano-structuring (e.g., 3DOM) provides a pathway to simultaneously enable an outstanding initial storage density (1706.4 kJ/kg) and exceptional cyclability (<6% loss after 125 cycles), its templated co-precipitation synthesis route suggests high manufacturing costs and low scalability. In comparison, redox systems have been less modified with nanomaterials to enhance kinetic performance. Still, existing studies indicate that nano-additives such as SiO_2_ and ZrO_2_ can significantly improve reversibility, reduce densification, and increase energy density (e.g., up to 364.98 kJ/kg for Co-based systems). However, the overall storage density of a redox system is relatively lower compared to the other thermochemical systems. Additionally, the high cost and toxicity issues for Co-based materials also remains a key challenge for practical application.

## 6. Limitations and Implementation Challenges for Nano-Engineered Composite Thermochemical Materials

### 6.1. Material-Level Limitations

**Chemical compatibility:** The high surface reactivity of nanoparticles, while beneficial for kinetics, can lead to undesirable side reactions at elevated temperatures. For instance, common doping agents like SiO_2_ and Al_2_O_3_ can react with CaO or Ca(OH)_2_ to form calcium silicates or aluminates (e.g., Ca_5_(SiO_4_)_2_(OH)_2_). These byproducts can irreversibly consume active material, reduce energy storage density, and alter the thermochemical properties of the composite over repeated cycles.

**Material cost:** The cost of high-performance nanomaterials (e.g., graphene, CNTs) and complex synthesis routes (e.g., sol–gel, templated co-precipitation for 3DOM structures) remains prohibitive for large-scale energy storage applications. While strategies using expanded graphite (EG) or nano-oxides are relatively cost-effective, applying them at high loadings (e.g., 30 wt% for flow enhancement) must be justified by sufficient improvements in performance and cycle life.

### 6.2. Pilot-Level Challenges

The transition from gram-scale laboratory testing to kilogram-scale pilot systems unveils critical challenges that cannot be observed in small material samples. These primarily revolve around reactor integration and rigorous validation under realistic conditions.

**Reactor-level performance:** At the pilot scale, overall system performance is governed not only by the material’s intrinsic properties but also critically by reactor-level engineering parameters. A primary challenge lies in the integration of these advanced materials into effective reactor configurations (e.g., fixed or fluidized beds), while achieving and maintaining stable and homogeneous fluidization without undesired effects such as agglomeration, sintering, deliquesces, and structural deformation. A uniform gas–solid contact throughout the reaction volume can effectively charge and discharge the system at high power densities. The reactor-level optimization is critical to fully leverage the enhanced kinetics and stability offered by nano-engineered materials at the system level.

**Long-term durability under realistic conditions:** A critical impediment to commercialization is the lack of robust validation of long-term durability at the pilot scale under realistic conditions. Conducting accelerated long-term durability testing (e.g., hundreds to thousands of cycles) at the pilot scale is essential to reliably quantify material degradation and identify degradation mechanisms, which is a critical step to reduce the major uncertainties before committing to commercial-scale deployment.

### 6.3. Commercialization Barriers

The final transition from validated pilot plants to commercially viable installations presents a distinct set of challenges centred on economics, manufacturing, and full-system engineering.

**Large-Scale Manufacturing:** Laboratory-scale methods (e.g., sol–gel, deposition–precipitation, templating) are often energy-intensive, involve complex synthesis steps, and precise conditions. Scaling these processes to an industrial level while preserving critical nano-structural properties (e.g., dopant dispersion, porous structure, interfacial bonding) is a considerable engineering challenge. The lack of commercially viable and scalable synthesis equipment also constitutes a major bottleneck for commercial applications.

**System Integration:** Engineering full-scale TCES plants (MWh scale) requires the integration and optimization of plant components and an advanced control system. Key challenges involve designing high-efficiency heat exchangers for rapid thermal cycling, developing gas–solid handling systems to maintain reactant flow and pressure, and implementing real-time control strategies for stable operation under dynamic conditions. Furthermore, material performance and system efficiency must be co-optimized to minimize energy losses and maximize overall efficiency. This requires a multidisciplinary approach integrating materials engineering with process design and automation.

**Cost-Effectiveness:** Low Levelized Cost of Storage (LCOS) is the key for large-scale deployment. Achieving reduced LCOS depends not only on the cost of advanced materials and their manufacturing, but also on their broader impact on downstream system expenses. This requires a comprehensive techno-economic evaluation to determine whether the performance benefits of advanced materials truly translate into overall cost savings at the system level.

## 7. Concluding Remarks

Thermochemical energy storage (TCES) has emerged as a critical solution for addressing the intermittency of renewable energy, offering high energy density, long-term stability, and minimal thermal losses. This review systematically examined the latest advancements in nano-engineered composite thermochemical materials (TCMs), highlighting how nano-structuring strategies, such as nanoparticle incorporation, porous matrices, flow-improving agents, and an engineered nanostructure, showing promising potential overcome the inherent limitations of conventional TCMs. Key findings of this work include the following:

- Sorption systems (e.g., salt hydrates) benefit from carbon-based nanomaterials (CNTs, graphene), which enhance both thermal conductivity and reaction kinetics, achieving energy densities up to 3935 kJ/kg with improved cycling stability.
- Hydroxides, redox, and carbonate systems leverage nanoparticle (e.g., SiO_2_, Al_2_O_3_, carbonaceous materials) doping and grain boundary engineering to mitigate sintering and agglomeration while enhancing reaction rates, thus enabling stable thermal cycles with minimal capacity loss. In addition, interfacial modification between reactants and nano-additives can further improve interfacial compatibility and dispersibility, contributing to the thermochemical storage performances.
- Nanoparticles can also serve as flowing agents to enhance the fluidization characteristics of bulk TCMs. This approach necessitates nanoparticle loadings as high as 30 wt.%, which presents significant limitations for practical thermochemical storage applications.
- Nanosized structure engineering presents another effective approach to improve thermochemical performance by increasing the reactants’ specific surface area and the associated reaction kinetics.

Despite the remarkable progress in laboratory-scale research, the practical deployment of nano-engineered TCMs faces several significant limitations and multi-scale challenges that must be addressed to bridge the gap between fundamental research and commercial application:

- The high surface reactivity of nanoparticles may lead to unintended chemical interactions with the TCMs, leading to side products that reduce the energy storage density and stability of thermal cycling. The chemical compatibility between the nanomaterials and the TCM should be carefully considered, especially at high temperature applications.
- Many nano-engineered TCMs rely on expensive nanomaterials (e.g., graphene) or complex synthesis methods (e.g., sol–gel, templated co-precipitation, CVD). Future work should prioritize low-cost nano-additives and scalable fabrication methods. In addition, emphasis should also be placed on optimizing nanoparticle loading levels to balance performance gains with cost-effectiveness.
- Most studies show material-level improvements; however, pilot-scale validation is still required for large-scale deployment. Future efforts must transition from gram-scale testing to integrated reactor demonstrations under realistic operating conditions to evaluate system-level performance, fluidization behaviour, heat transfer efficiency, and gas–solid interaction.
- Current studies lack comprehensive techno-economic assessments of nano-engineered TCES systems, particularly in evaluating their levelized cost of storage (LCOS). Such assessments are essential in the future to identify cost bottlenecks, optimize material and reactor designs, and establish a viable commercialization pathway that meets grid-scale storage cost targets.

In summary, nanotechnology provides powerful tools to overcome intrinsic material limitations of different classes of TCMs. Future research must transfer from purely material-centric studies to system engineering, prioritizing scalable synthesis, pilot-scale validation, and thorough techno-economic analysis.

## Figures and Tables

**Table 1:** Comparison between different sorption TCMs [56].

Category	Solid Physical Adsorbents	Solid Chemical Adsorbents	Liquid Absorption
Mechanism	Physical interaction between water vapour and porous solids	Reversible chemical reactions forming salt hydrates	Concentration changes in liquid solutions
Materials	zeolites, silica gels, and activated carbon, MOF	CaCl_2_, MgSO_4_, MgCl_2_, MgSO_4_, SrBr_2_	LiCl solution, LiBr solution
Energy Density (kwh/m^3^)	50–220	800–1200	250–300
charging temperature (°C)	50–150	30–200	15–100
Advantages	Strong water affinity; High discharge temp (>55 °C)	Highest energy density Wide operational temperature range Moderate charging temperatures	Utilization of low-grade heat High TRL level
Challenges	High charging temps (100–250 °C) Low energy density Volume expansion/contraction issue	Poor cyclability Agglomeration Deliquescence	Few material pairs Corrosion issue

**Table 2:** Properties and costs of salt hydrates TCMs [53,56,83,84].

Sorbent	DHR at 25 °C	Charging Temperature (°C)	Heat of Reaction (kwh/m^3^)	Price (EUR/MJ)
CaCl_2_·6H_2_O	28–29%	45~138	120~381	0.1
LiCl·H_2_O	11%	66~87	253~400	9.11
LiBr·2H_2_O	7%	40~90	252~313	55
SrBr_2_·6H_2_O	60% (30 °C)	80	60~321	4.13
CaSO_4_·2H_2_O	/	--	390	/
MgCl_2_·6H_2_O	30–35%	130~150	556~695	0.09
MgSO_4_·7H_2_O	87–89%	122~150	400~924	0.07
Na_2_S·5H_2_O	34%	80~95	780	/

**Table 3:** Performance and cost overview of nano-engineered thermochemical materials.

Materials	Nano-Additives	Preparation Methods	Key Performance Metrics	Major Challenges	Scalability
Sorption-based system	Carbon additives (CNTs, graphene, activated carbon, Carbon nanospheres, EG)	Mechanical mixing Impregnation method	Energy density: up to 3935 kJ/kg Enhanced kinetics due to high specific surface area and hydrophilic groups Enhanced thermal conductivity	Deliquescence Cost of nanomaterials Lack of device level evaluation	Medium Low-cost salt hydrates but expensive nanomaterials (e.g., Graphene, CNTs) Lack of large-scale validation
Hydroxide system	EG, nano-porous, CNTs, SiO_2_, Al_2_O_3_	Deposition–Precipitation Impregnation method Mechanical Mixing Wet coating Microwave hydrothermal method	Energy density: ~1300 kJ/kg Improved hydration kinetics Sintering resistance Enhanced cyclic stability	Side reactions (e.g., Ca_5_(SiO_4_)_2_(OH)_2_) Interface compatibility High loading (30 wt%) of nano-additives increases cost	Medium to high Low cost metal hydroxides EG and oxides (SiO_2_, Al_2_O_3_) are relatively low-cost Device level validation
Carbonate system	SiO_2_, Al_2_O_3_, graphite, TiO_2_, MgO, Mn and Zr doping	Mechanical Mixing Chemical vapour deposition Templated co-precipitation method	Energy density: ~1313 kJ/kg (50 cycles) Sintering resistance Lower decarbonation temperature Enhanced cyclic stability	Side reactions Capacity fade over cycles Formation of inert phases	Low to Medium Low cost metal carbonates Low-cost oxide additives (e.g., SiO_2_) Complex synthesis
Redox system	SiO_2_, ZrO_2_	Sol–gel approach Dry mixing Solid-phase calcination	Energy density: 364.98 J kJ/kg Improved reversibility Reduced densification	Cost and toxicity (Co-based) Sintering-induced degradation Incomplete conversion	Low Cost and toxicity of Co-based oxides Relatively low reaction enthalpy
